# Evaluation of the accuracy and readability of ChatGPT-4 and Google Gemini in providing information on retinal detachment: a multicenter expert comparative study

**DOI:** 10.1186/s40942-024-00579-9

**Published:** 2024-09-02

**Authors:** Piotr Strzalkowski, Alicja Strzalkowska, Jay Chhablani, Kristina Pfau, Marie-Hélène Errera, Mathias Roth, Friederike Schaub, Nikolaos E. Bechrakis, Hans Hoerauf, Constantin Reiter, Alexander K. Schuster, Gerd Geerling, Rainer Guthoff

**Affiliations:** 1 Department of Ophthalmology, Medical Faculty and University Hospital Düsseldorf – Heinrich Heine University Düsseldorf, Düsseldorf, Germany; 2 UPMC Eye Center, University of Pittsburgh, Pittsburgh, PA USA; 3 Department of Ophthalmology, University Hospital of Basel, Basel, Switzerland; 4 Department of Ophthalmology, University Medical Centre Rostock, Rostock, Germany; 5 Department of Ophthalmology, University Hospital Essen, Essen, Germany; 6 Department of Ophthalmology, University Medical Center Göttingen, Göttingen, Germany; 7 Department of Ophthalmology, Helios HSK Wiesbaden, Wiesbaden, Germany; 8 Department of Ophthalmology, Mainz University Medical Centre of the Johannes Gutenberg, University of Mainz, Mainz, Germany

**Keywords:** Retinal detachment, ChatGPT-4, Google Gemini, Artificial intelligence, Language learning models, Patient education

## Abstract

**Background:**

Large language models (LLMs) such as ChatGPT-4 and Google Gemini show potential for patient health education, but concerns about their accuracy require careful evaluation. This study evaluates the readability and accuracy of ChatGPT-4 and Google Gemini in answering questions about retinal detachment.

**Methods:**

Comparative study analyzing responses from ChatGPT-4 and Google Gemini to 13 retinal detachment questions, categorized by difficulty levels (D1, D2, D3). Masked responses were reviewed by ten vitreoretinal specialists and rated on correctness, errors, thematic accuracy, coherence, and overall quality grading. Analysis included Flesch Readability Ease Score, word and sentence counts.

**Results:**

Both Artificial Intelligence tools required college-level understanding for all difficulty levels. Google Gemini was easier to understand (*p* = 0.03), while ChatGPT-4 provided more correct answers for the more difficult questions (*p* = 0.0005) with fewer serious errors. ChatGPT-4 scored highest on most challenging questions, showing superior thematic accuracy (*p* = 0.003). ChatGPT-4 outperformed Google Gemini in 8 of 13 questions, with higher overall quality grades in the easiest (*p* = 0.03) and hardest levels (*p* = 0.0002), showing a lower grade as question difficulty increased.

**Conclusions:**

ChatGPT-4 and Google Gemini effectively address queries about retinal detachment, offering mostly accurate answers with few critical errors, though patients require higher education for comprehension. The implementation of AI tools may contribute to improving medical care by providing accurate and relevant healthcare information quickly.

## Background

Our clinical practice has already been transformed by the internet over the last few decades [1]. In particular, recently introduced data-driven tools such as artificial intelligence (AI) have the potential to revolutionize healthcare even more in the future [2–4]. This change is already underway, with more people turning to online platforms and self-diagnosis tools, such as symptom checkers [5] for healthcare information [6, 7], particularly as accessing traditional face to face medical care becomes more challenging. However, these online tools often lack essential details to accurately assess symptom urgency [7]. Yet, there may be a shift on the horizon. Recent initiatives by the World Health Organization (WHO) seek to set standards for AI-assisted healthcare technologies, encouraging additional exploration of their potential benefits [8].

Large language models (LLM) like ChatGPT-4 launched for public use in November 2022 and Google Gemini, released in December 2023 and renamed in February 2024 also offer advantages in patient health’s education [9]. However, there are concerns that while they can write persuasive texts, these can potentially be inaccurate, distorting scientific facts and spreading misinformation [9].

Providing accurate and timely healthcare information is critical in a serious eye condition that requires immediate treatment, such as acute retinal detachment (RD) or endophthalmitis. Prompt treatment is essential to reduce the risk of permanent visual deterioration, as duration of macula-involving RD is one of the few modifiable factors for a better postoperative visual outcome [10]. Patients with acute RD often seek medical care sooner, are more conscious of the symptoms of RD [11], and tend also to be better educated [12]. This suggests that raising awareness and educating patients about the classic signs of RD could not only result in more patients seeing an ophthalmologist while their macula is still attached but could also result in a better postoperative outcome for patients.

The aim of this study is to evaluate the readability and accuracy of ChatGPT-4 and Google Gemini in responding to queries about RD.

## Methods

In our comparative study, we included 13 questions frequently asked by patients on topics such as symptoms, causes of retinal detachment, surgical techniques and follow-up treatment. These questions were categorized into three difficulty levels (D1-D3) by two vitreoretinal specialists (P.S. and R.G.) (Table 1).

**Table 1:** All 13 questions sorted by difficulty level

Question	Difficulty level 1
Q1	I see a shadow. What should I do?
Q2	I see flashes of light. What should I do?
Q3	I see floaters in one eye. What should I do?
	**Difficulty level 2**
Q4	What are the risk factors for retinal detachment?
Q5	What forms of retinal detachment are there?
Q6	How does a retinal detachment develop?
Q7	How quickly does a retinal detachment need to be treated?
Q8	What are the chances of success of vitrectomy for retinal detachment?
	**Difficulty level 3**
Q9	What are the treatment options for retinal detachment?
Q10	How exactly is a vitrectomy performed to treat a retinal detachment?
Q11	Which tamponades are used in vitrectomy for retinal detachment?
Q12	How do gas tamponades differ from silicone oil tamponades in retinal surgery?
Q13	What needs to be considered during postoperative care after vitrectomy?

To obtain the most precise and specialized answer possible, ChatGPT-4 (Generative Pre-trained Transformer), developed by OpenAI (San Francisco, CA, USA) and Google Gemini (Google DeepMind, London, United Kingdom) were instructed via a prompt to assume the role of an ophthalmologist when answering:

*Take the role of an ophthalmologist who answers patients’ questions. Write in continuous text and exclude images and illustrations for explanation. Your task is to give a concise*,* specific answer that is accurate by current standards. The length of the answer should not exceed 150 words.*

Each question was asked independently in a new chat window after the prompt was repeated, and the previous dialogue was deleted to avoid any possible interference of the previous questions and answers with the following ones. The evaluation criteria included the correctness, errors, thematic accuracy and coherence of the answers.

### Evaluation of the answers

The answer options for each question in the online survey were organized as follows:

#### Correctness (single answer)

- Correct: The entire answer is correct.
- Partially incorrect: The core statement of the answer is correct, but the rest of the answer contains one or more errors.
- Incorrect: The core statement of the answer is incorrect.

#### Error rating if applicable (multiple answers)

- Serious errors in content: The core statement of the answer AND / OR the rest of the answer contains one or more serious errors in content that could have serious consequences / pose a risk to patients.
- Content errors: The core statement of the answer contains one or more content errors that do not pose a risk to patients OR the core statement of the answer is correct, but the rest of the answer contains one or more content errors that do not or only slightly change the core statement of the answer and do not pose a risk to patients.
- Formal errors: The answer contains one or more grammatical or linguistic errors, for example, but these do not affect the core message of the answer or have any other significant consequences.

#### Thematic accuracy (single answer)

- Applicable: The answer identifies the central concept and is thematically specific.
- Partially correct: The answer identifies the central concept, but also partially addresses an unrelated topic.
- Not applicable: The answer does not identify the central concept and / or targets an unrelated topic.

#### Coherence (single answer)

- Coherent: The core message of the answer is fully supported by the rest of the answer.
- Partially coherent: The core statement of the answer is essentially confirmed by the rest of the answer, but there are deviating statements / contradictions in the rest of the answer.
- Incoherent: The core statement of the answer contradicts the rest of the answer.

For the parameter’s correctness, thematic accuracy and coherence, only a single answer was possible; for error assessment, multiple answers or assessments of individual parts of the answer were possible due to the different error categories (content vs. formal errors).

Our 13 masked questions and the corresponding answers from ChatGPT-4 and Google Gemini were sent online to ten experienced vitreoretinal specialists via the RedCap platform [13, 14].

Each question was given an overall quality grading at the end in addition to the assessment of the correctness, accuracy, thematic accuracy and coherence of the answers. The overall quality grades were categorized based on the American GPA scoring system as follows: excellent = 4 points, good = 3 points, satisfactory = 2 points, sufficient = 1 points, bad = 0 points [15].

### Evaluation of readability

The readability of all generated answers was analyzed with the online tool readable (Readable.com, Horsham, United Kingdom) with regard to number of words, number of sentences, number of words per sentence, number of long words (> 6 letters), Flesch Reading Ease (FRES) score [16] and reading level.

The formula for calculating the FRES is:

206.835 − 1.015 × (Total Words / Total Sentences) − 84.6 × (Total Syllables / Total Words).

Flesch Readability Ease Score for evaluating the readability of a text is shown in Table 2.

**Table 2:** The table shows the FRE score with corresponding school level and description of the reading difficulty level [17]

FRE score	School Level	Interpretation
100.0–90.0	5th Grade	Very easy to read. Easily understood by an average 11-year-old student.
90.0–80.0	6th Grade	Easy to read. Conversational English for consumers.
80.0–70.0	7th Grade	Fairly easy to read
70.0–60.0	8th-9th Grade	Plain English. Easily understood by 13- to 15-year-old students
60.0–50.0	10th-12th Grade	Fairly difficult to read.
50.0–30.0	College	Difficult to read
30.0–10.0	College Graduate	Very difficult to read. Best understood by university graduates
10.0–0.0	Professional	Extremely difficult to read. Best understood by university graduates

### Statistical analysis

Statistical analysis was performed using GraphPad Prism10, Version 10.2.2 (341), (GraphPad Software, San Diego, USA) for Mac. For statistical analysis, categorical variables were presented as absolute and relative frequencies, whereas mean and standard deviation were computed for approximately normal-distributed continuous variables, otherwise median and interquartile range. Evaluation of data normality was performed using the Shapiro-Wilk test. Welch’s t-test was used to evaluate the difference in means in both Large Language Models. Fisher’s Exact Test was used to evaluate the association between categorical variables. Non-normally distributed continuous variables were compared using Mann-Whitney test. For multiple comparisons, Brown-Forsythe and Welch ANOVA test or non-parametric Kruskal-Wallis test and post hoc Dunn’s test with correction for multiple testing were used. All statistical tests were two-sided and *p*-value < 0.05 was considered statistically significant.

### Ethical considerations

In concordance with German legislation, an approval of a medical ethical committee was not needed for a study that did not include patient data. The study was performed in accordance with the ethical standards set forth in the 1964 Declaration of Helsinki.

## Results

### Readability

#### Flesch Readability ease score (FRES)

**Fig. 1:**
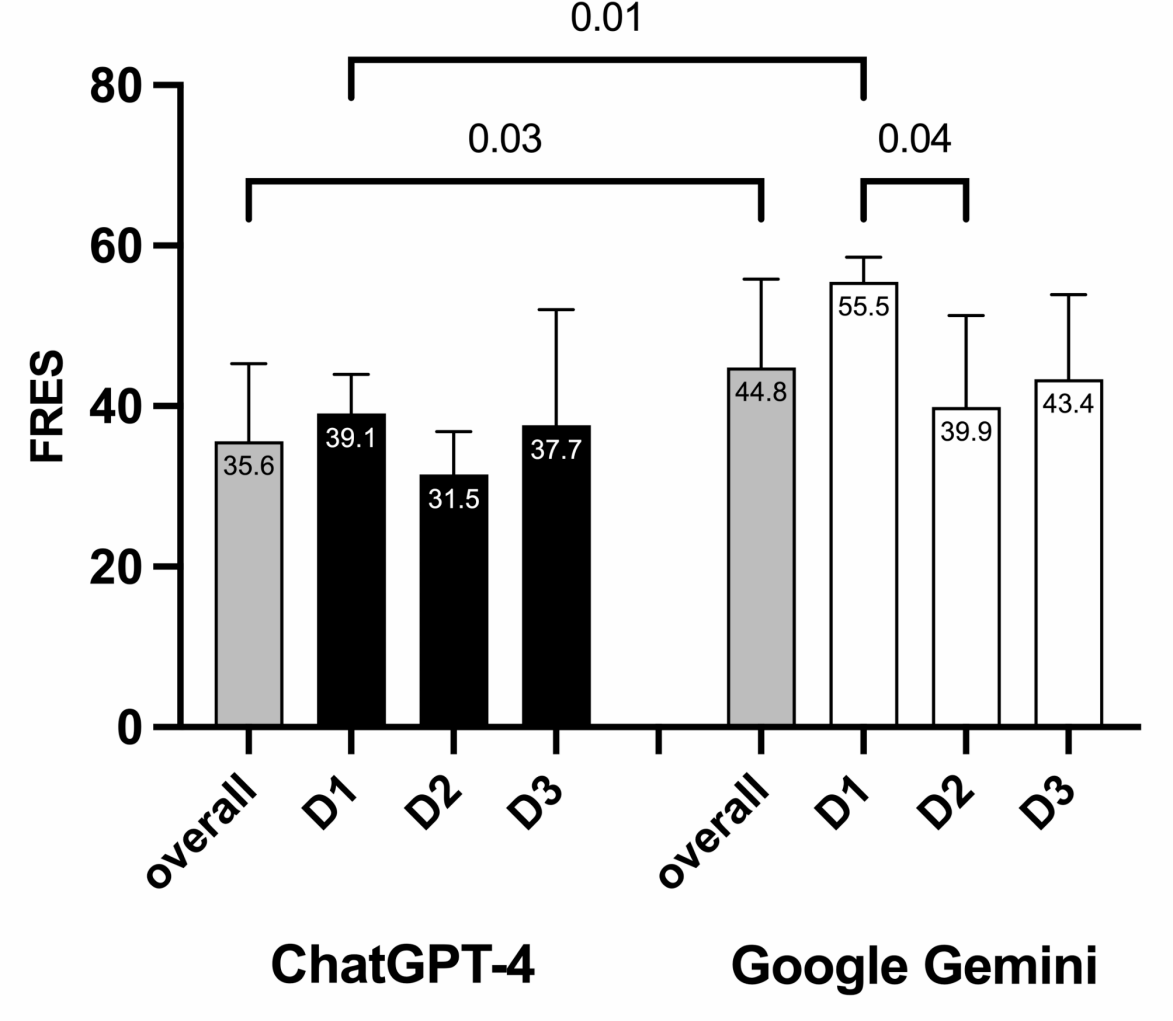
Flesch Readability Ease Score (FRES) for ChatGPT-4 and Google Gemini overall and for all difficulty levels (D1, D2, D3). The bars represent the mean FRES values, and the whiskers indicate the standard deviation (SD)

The overall FRES was 36 ± 9.7 for ChatGPT-4 and 45 ± 11 for Google Gemini (*p* = 0.03). Regarding the level of difficulty for D1 (low) there was a significantly lower FRES for ChatGPT-4 39.1 ± 4.9 compared to 55.5 ± 3.1 for Google Gemini (*p* = 0.01). For D2 (medium) and D3 (high) the FRES was 31.5 ± 5.4 and 39.9 ± 11.4 (*p* = 0.2) and 37.7 ± 14.4 and 43.4 ± 10.5 (*p* = 0.5) for ChatGPT-4 and Google Gemini, respectively. While no statistically significant difference in FRES was found for D1, D2 and D3 for ChatGPT-4, a significant difference was found between D1 and D2 for Google Gemini (*p* = 0.04) (Fig. 1).

#### Number of words

The mean number of words was 159 ± 20.6 and 155 ± 42.3 for ChatGPT-4 and Google Gemini, respectively (*p* = 0.76). Answers generated by ChatGPT-4 for D1, D2 and D3 counted 179 ± 39.1, 150 ± 6.7 and 156 ± 7.7 words (*p* = 0.17). For Google Gemini the mean number of words in D1, D2 and D3 was 155 ± 20.8, 115 ± 14.3 (*p* = 0.05) and 195 ± 31.3 (*p* = 0.003). The mean difference in number of words was significant between ChatGPT-4 and Google Gemini for D2 (+ 34.6 words, *p* = 0.003) and D3 (-38.4 words, *p* = 0.04).

#### Number of sentences

The mean number of sentences was 9.1 ± 1.9 and 8.7 ± 3.2 for ChatGPT-4 and Google Gemini, respectively (*p* = 0.72). Answers generated by ChatGPT-4 for D1, D2 and D3 showed no significant difference in 8.7 ± 3.2, 8.8 ± 1.9 and 9.6 ± 1.5 sentences (*p* = 0.67). In contrast, for Google Gemini the mean number of sentences in D1, D2 and D3 was 7.3 ± 1.5, 6.0 ± 1.0 (*p* = 0.01) and 12.2 ± 1.9 (*p* = 0.0007). The mean difference in number of sentences was significant between ChatGPT-4 and Google Gemini for D2 (+ 2.8 sentences, *p* = 0.03) and D3 (-2.6 sentences, *p* = 0.047).

#### Number of words per sentence

The mean number of words per sentence was 18.3 ± 4.2 for ChatGPT-4 and 18.6 ± 3.1 for Google Gemini (*p* = 0.76). Answers generated by ChatGPT-4 for D1, D2 and D3 counted 21.8 ± 5.7, 17.7 ± 4.4 and 16.7 ± 2.8 words (*p* = 0.21). For Google Gemini the mean number of words in D1, D2 and D3 was 21.3 ± 1.9, 19.5 ± 3.3 (*p* = 0.35) and 16.0 ± 1.2 (*p* = 0.02).

#### Number of long words

The mean number of long words (more than 6 letters) was 34.3 ± 4.5 and 29.7 ± 7.0 for ChatGPT-4 and Google Gemini, respectively (*p* = 0.76). Answers generated by ChatGPT-4 for D1, D2 and D3 counted 31.1 ± 3.0, 35.7 ± 5.0 and 34.8 ± 4.4 words (*p* = 0.17). For Google Gemini the mean number of long words in D1, D2 and D3 was 24.4 ± 2.4, 30.0 ± 8.1 (*p* = 0.05) and 32.6 ± 6.9 (*p* = 0.003). The mean difference in the number of long words was significant between ChatGPT-4 and Google Gemini for D1 (+ 6.7 words, *p* = 0.04).

### Correctness

For the difficulty level 1 and 2, there was no significant difference between ChatGPT-4 and Google Gemini in terms of correctness (*p* = 0.5). The total number of correct versus partially correct answers in difficulty level 3 was 36 vs. 13 for ChatGPT-4 and 18 vs. 30 for Google Gemini (*p* = 0.0005) (Table 3).

**Table 3:** Correctness - number of correct, partially correct and incorrect answers for all 13 questions and difficulty levels

		Correctness						
		ChatGPT-4			Google Gemini			
Question	Difficulty level	**Correct**	**Partially correct**	**incorrect**	**Correct**	**Partially correct**	**incorrect**	***p*** **-value**
1	D1	9	1	0	7	2	1	0.5
2		10	0	0	9	1	0	0.9
3		10	0	0	10	0	0	1
	D1 total	29	1	0	26	3	1	0.4
4	D2	8	2	0	10	0	0	0.5
5		7	3	0	7	3	0	1
6		6	3	1	7	2	1	0.9
7		5	4	1	4	6	0	0.5
8		9	1	0	4	5	1	0.06
	D2 total	35	13	2	32	16	2	0.7
9	D3	8	2	0	1	9	0	0.005
10		10	0	0	5	4	1	0.04
11		5	4	1	2	7	1	0.4
12		4	6	0	4	6	0	1
13		9	1	0	6	4	0	0.3
	D3 total	36	13	1	18	30	2	0.0005

### Errors (multiple answers possible)

The number of serious errors was higher for all difficulty levels in Google Gemini compared to ChatGPT-4 (D1: 1 vs. 0; D2: 4 vs. 2; D3: 4 vs. 1). Google Gemini also showed more errors in terms of content (D1: 3 vs. 1; D2: 14 vs. 12; D3: 21 vs. 9, *p* = 0.03) (Table 4).

**Table 4:** Errors - number of serious errors, content and formal errors for all 13 questions and difficulty levels

		Errors (multiple answers possible)						
		ChatGPT-4			Google Gemini			
Question	Difficulty level	**Serious errors**	**Content errors**	**Formal errors**	**Serious errors**	**Content errors**	**Formal errors**	***p*** **-value**
1	D1	0	1	0	1	2	0	0.9
2		0	0	0	0	1	0	-
3		0	0	0	0	0	0	-
	D1 total	0	1	0	1	3	0	0.9
4	D2	0	2	1	0	0	0	-
5		0	3	1	0	3	0	0.9
6		1	2	2	1	2	0	0.8
7		1	4	0	1	5	0	0.9
8		0	1	0	2	4	0	0.9
	D2 total	2	12	4	4	14	0	0.7
9	D3	0	2	0	1	8	0	0.9
10		0	0	0	1	4	0	-
11		0	3	0	0	4	0	0.9
12		1	3	0	1	2	0	0.9
13		0	1	0	1	3	0	0.9
	D3 total	1	9	0	4	21	0	0.03

### Thematic accuracy and coherence

The thematic accuracy (Table 5) and coherence (Table 6) showed higher scores for ChatGPT-4 compared to Google Gemini in terms of difficulty level 3 (*p* = 0.003), whereas there was no statistically significant difference for both LLMs in difficulty level 1 and 2.

**Table 5:** Thematic accuracy - number of applicable, partially applicable and not applicable answers for all 13 questions and difficulty levels

		Thematic accuracy						
		ChatGPT-4			Google Gemini			
Question	Difficulty level	**Applicable**	**Partially applicable**	**Not applicable**	**Applicable**	**Partially applicable**	**Not applicable**	***p*** **-value**
1	D1	8	2	0	8	2	0	1
2		10	0	0	7	3	0	0.2
3		9	1	0	9	1	0	1
	D1 total	27	3	0	24	6	0	0.5
4	D2	9	1	0	9	1	0	1
5		9	1	0	9	1	0	1
6		4	6	0	9	1	0	0.06
7		8	2	0	5	5	0	0.4
8		8	2	0	8	2	0	1
	D2 total	38	12	0	40	10	0	0.8
9	D3	10	0	0	6	4	0	0.09
10		9	1	0	7	3	0	0.6
11		10	0	0	8	2	0	0.5
12		9	1	0	9	1	0	1
13		10	0	1	6	4	0	0.05
	D3 total	48	2	1	36	14	0	0.003

**Table 6:** Coherence - number of coherent, partially coherent and incoherent answers for all 13 questions and difficulty levels

		Coherence						
		ChatGPT-4			Google Gemini			
Question	Difficulty level	**Coherent**	**Partially coherent**	**Incoherent**	**Coherent**	**Partially coherent**	**Incoherent**	***p*** **-value**
1	D1	9	1	0	7	3	0	0.58
2		10	0	0	9	1	0	0.9
3		8	2	0	9	1	0	0.9
	D1 total	27	3	0	25	5	0	0.7
4	D2	7	3	0	10	0	0	0.2
5		10	0	0	9	1	0	0.9
6		4	6	0	10	0	0	0.01
7		7	2	1	6	4	0	0.7
8		10	0	0	4	6	0	0.01
	D2 total	38	11	1	39	11	0	0.9
9	D3	10	0	0	7	3	0	0.2
10		9	1	0	7	3	0	0.6
11		9	1	0	9	1	0	1
12		8	2	0	8	2	0	1
13		9	1	0	7	3	0	0.6
	D3 total	45	5	0	38	12	0	0.1

### Overall quality grading for each question

ChatGPT-4 outperformed Google Gemini in 8 out of 13 (62%) questions. Significantly better grades were achieved in Q1 3.5 ± 0.7 vs. 2.1 ± 0.9 (*p* = 0.001), Q2 3.7 ± 0.5 vs. 2.6 ± 0.7 (*p* = 0.01), Q8 3.3 ± 0.7 vs. 1.7 ± 1.1 (*p* = 0.001), Q9 3.3 ± 0.7 vs. 1.7 ± 1.3 (*p* = 0.002), Q10 3.4 ± 0.5 vs. 2.1 ± 1.1 (*p* = 0.005) and Q13 3.2 ± 1.3 vs. 2.0 ± 1.2 (*p* = 0.005) for ChatGPT-4. In comparison, Google Gemini achieved significant higher scores only in Q6 3.1 ± 0.9 vs. 1.8 ± 0.9 (*p* = 0.004).

### Overall quality grading vs. difficulty level

**Fig. 2:**
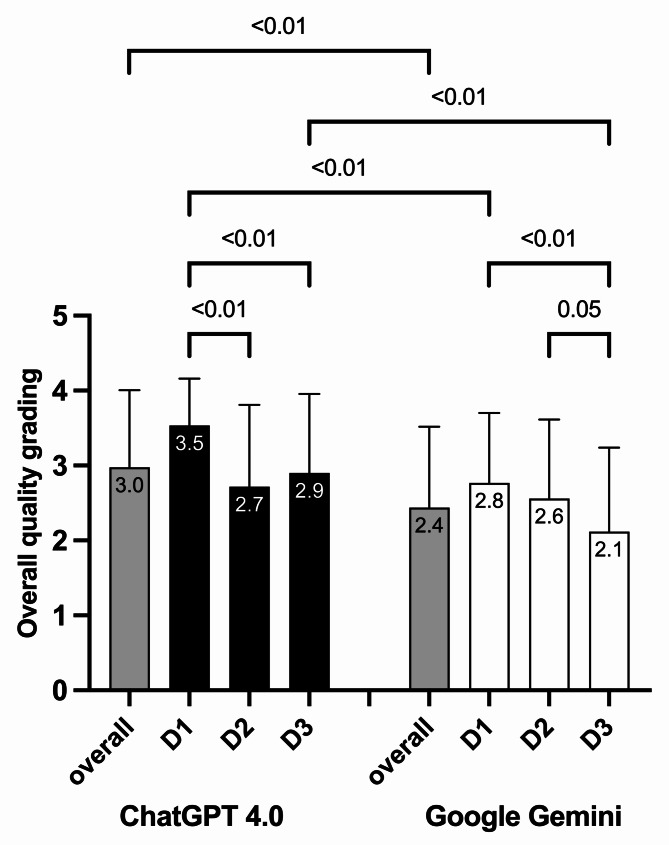
shows the quality grading in relation to the difficulty level (D1, D2, D3) for ChatGPT-4 and Google Gemini. The bars represent the mean quality grading values, and the whiskers indicate the standard deviation (SD)

The overall quality grading was significantly higher for ChatGPT-4 compared to Google Gemini (3.0 ± 1.0 vs. 2.4 ± 1.1, respectively; *p* < 0.01). In terms of difficulty level D1, ChatGPT-4 scored 3.5 ± 0.6 significantly better compared to 2.8 ± 0.9 for Google Gemini (*p* < 0.01). There was no significant difference between ChatGPT-4 2.7 ± 1.1 and Google Gemini 2.6 ± 1.1 for D2. For D3, ChatGPT-4 received better grades 2.9 ± 1.1 than Google Gemini 2.1 ± 1.1 (*p* < 0.01). In addition, both ChatGPT-4 (D1: 3.5 ± 0.6; D2: 2.7 ± 1.1; D3 2.9 ± 1.1; *p* < 0.01) and Google Gemini showed significantly lower grades as the difficulty level increased (D1: 2.8 ± 0.9; D2: 2.6 ± 1.1; D3: 2.1 ± 1.1; *p* < 0.01) (Fig. 2).

## Discussion

Retinal detachment (RD) is a sight-threatening eye condition that requires immediate surgical intervention to prevent permanent visual impairment. Providing timely and accurate health information is critical to patient understanding and treatment outcomes [10, 12]. In our study, ChatGPT-4 and Google Gemini showed promise in answering typical patient questions about RD. They delivered mostly correct and accurate responses with few serious errors. However, a college-level education is needed to comprehend the answers across various difficulty levels.

Large language models (LLMs) such as ChatGPT-4 and Google Gemini can provide health-related information to the users [18]. ChatGPT-4 is an autonomous machine-learning system capable of quickly generating complex and seemingly intelligent text in a conversational style in multiple languages, including English [9, 19]. Key benefits include its accessibility, cost-free usage, user-friendliness, and ongoing enhancements [9]. Consequently, it is conceivable that ChatGPT-4 could be used to help patients answer their health questions. The ability of ChatGPT-4 to respond to questions about medical examinations, including those related to ophthalmology [20, 21], has been the subject of great interest and has been investigated in several studies [22, 23].

In our study, based on the Flesch Readability Ease Score (FRES), both ChatGPT-4 and Google Gemini required at least a university degree to understand the answers, regardless of the difficulty level of the questions (D1-D3). However, Google Gemini was found to be easier to understand than ChatGPT-4, with scores of 45 ± 11 vs. 36 ± 9.7, respectively (*p* = 0.03). This trend persisted for D1 questions separately, with scores of 55.5 ± 3.1 for Google Gemini vs. 39.1 ± 4.9 for ChatGPT-4 (*p* = 0.01). For more challenging D2-D3 questions, there was no significant difference between Google Gemini and ChatGPT-4, with scores of 39.9 ± 11.4 vs. 31.5 ± 5.4 (*p* = 0.2) and 43.4 ± 10.5 vs. 37.7 ± 14.4 (*p* = 0.5), respectively.

Both LLMs were instructed to provide answers of up to 150 words in length. However, the mean number of words exceeded this limit, with an average of 159 ± 20.6 for ChatGPT-4 and 155 ± 42.3 for Google Gemini. Regarding the mean number of sentences, there was no significant difference between both models, with averages of 9.1 ± 1.9 for ChatGPT-4 and 8.7 ± 3.2 for Google Gemini (*p* = 0.72). LLMs can exceed the word limits suggested in the prompts for several reasons. They interpret prompts based on patterns from their training data, which may include longer responses. In particular, different text lengths in the training data can explain this behavior. Complex prompts may also require detailed explanations, leading to longer responses. Ambiguity in the instructions and the model’s goal of providing relevant and coherent responses can also lead to exceeding the limit. Interestingly, Google Gemini required more sentences for the more difficult questions, with averages of 7.3 ± 1.5 for D1 and 12.2 ± 1.9 for D3 (*p* = 0.0007). There was no difference between ChatGPT-4 and Google Gemini concerning the mean number of words per sentence. It was 18.3 ± 4.2 for ChatGPT-4 and 18.6 ± 3.1 for Google Gemini on average. The mean number of long words (defined as those with more than 6 letters) was 34.3 ± 4.5 for ChatGPT-4 and 29.7 ± 7.0 for Google Gemini (*p* = 0.76). The mean difference in the number of long words was significant between both AI tools for D1, with ChatGPT-4 exhibiting a higher count by 6.7 words on average (*p* = 0.04).

In terms of correctness, for the D1 and D2 questions, there was no significant difference between ChatGPT-4 and Google Gemini (*p* = 0.5). For D3, the total number of correct versus partially correct answers was 36 vs. 13 for ChatGPT-4 and 18 vs. 30 for Google Gemini (*p* = 0.0005). However, it is important to note that opinions on specific retinal disease treatments may vary, even among retinal specialists, and thus may affect the analysis of correctness. The number of serious errors was altogether low, but higher for all difficulty levels in Google Gemini compared to ChatGPT-4 (D1: 1 vs. 0; D2: 4 vs. 2; D3: 4 vs. 1). In terms of thematic accuracy and coherence, ChatGPT-4 showed higher scores compared to Google Gemini in terms of high difficulty level (*p* = 0.003), whereas there was no statistically significant difference for both LLMs in low and medium difficulty levels.

Considering the overall quality grades for each question, ChatGPT-4 outperformed Google Gemini in 8 out of 13 questions. In addition, ChatGPT-4 received better grades in difficulty levels D1 and D3: 3.5 ± 0.6 compared to 2.7 ± 0.9 (*p* = 0.002) and 2.9 ± 1.1 compared to 2.1 ± 1.1 (*p* = 0.0002), respectively. In addition, both ChatGPT-4 (*p* = 0.007) and Google Gemini (*p* = 0.02) achieved significantly lower grades with increasing difficulty.

Public health professionals should pay attention to online health-seeking behaviors, weighing potential benefits, addressing quality concerns, and outlining criteria for evaluation of online health information [24].

More than one-third of adults in the United States routinely use the internet for self-diagnosis, for both non-urgent and urgent symptoms [6, 7] Patients search for information via search engines like Google or Yahoo, as well as on health websites. This can help individuals to gain a deeper understanding of medical conditions alongside professional healthcare advice [25]. However, the popular symptom-related websites of the major search engines often lack most of the information needed to make a decision about whether a particular symptom requires immediate medical attention [7].

Misdiagnosis by physicians occurs in approximately 5% of outpatients [26]. In a study with a total of 118 physicians in the US correctly diagnosed 55.3% of easier and 5.8% of more difficult cases (*p* < 0.001) [27]. When asked about the accuracy of their initial diagnosis received via Symptom Checker, 41% of patients said that a doctor had confirmed their diagnosis and 35% said that they had not seen a doctor for a professional assessment [6]. An evaluation of 23 known symptom checker apps found that an appropriate categorization recommendation was made in 80% of emergencies, a rate comparable to doctors in training and nurses in training [27]. An AI system known as Babylon AI, which is used in Africa for triage and diagnostic purposes, has shown that it is able to recognize the disease presented in a clinical case with an accuracy comparable to that of human doctors [28].

Importantly, ChatGPT-4, like other LLMs can generate persuasive and subtle [29] but often inaccurate text, sometimes referred as a ‘hallucination’ [30] leading to the distortion of scientific facts and the spread of misinformation [9]. Importantly, the content of LLMs needs to be reviewed [29]. Future discussion should focus on the how rather than the if of introducing this technology [19].

Our study has certain limitations. We only used the two best known LLMs to assess the questions. Further validation with multiple LLMs is needed. We only included the most common questions asked by patients, but this may not fully reflect the complexity of patient education. In addition, treatment recommendations may also vary between different ophthalmologists. Human-generated responses may also generate controversial opinions and should be further investigated in subsequent studies. In addition, the study is limited to the English language, which may not take into account the different levels of education and understanding of patients in other languages. We also did not address potential accessibility issues, such as visual impairment, that may hinder access to AI-based tools. In addition, the instructions were specific to the LLMs, which may not fully reflect how patients would utilize such technology.

## Conclusions

To summarize, ChatGPT-4 and Google Gemini showed promise in answering questions about retinal detachment, providing mostly correct answers with few critical errors, even though patients need higher education with good reading comprehension to understand them. The use of AI tools may help to improve medical care by providing accurate and relevant health information quickly. Based on the results of our study, LLMs show promise but are not yet suitable as a sole resource for patient education due to the risk of critical errors. We would suggest using these AI tools as supplementary rather than primary sources of information until further improvements are made to minimize errors and improve accessibility for a wider patient population.

## Data Availability

The datasets used and/or analyzed during the current study are available from the corresponding author on reasonable request.

## Abbreviations

AI: Artificial intelligence
FRES: Flesch Readability Ease Score
GPT: Generative Pre-training Transformer
LLMs: Large Language Models
WHO: World Health Organization
